# Deletion of *Asrgl1* Leads to Photoreceptor Degeneration in Mice

**DOI:** 10.3389/fcell.2021.783547

**Published:** 2022-01-18

**Authors:** Yu Zhou, Wanli Tian, Xiaoyan Jiang, Huining Yang, Zhilin Jiang, Xiao Li, Dan Jiang, Kuanxiang Sun, Yeming Yang, Wenjing Liu, Xianjun Zhu

**Affiliations:** ^1^ Sichuan Provincial Key Laboratory for Human Disease Gene Study, Department of Laboratory Medicine, Center for Medical Genetics, Sichuan Provincial People’s Hospital, School of Medicine, University of Electronic Science and Technology of China, Chengdu, China; ^2^ Research Unit for Blindness Prevention of the Chinese Academy of Medical Sciences (2019RU026), Sichuan Academy of Medical Sciences and Sichuan Provincial People’s Hospital, Chengdu, China; ^3^ Department of Ophthalmology, First People’s Hospital of Shangqiu, Shangqiu, China

**Keywords:** retinitis pigmentosa, asparaginase and isoaspartyl peptidase 1, knockout mouse model, RNA-seq, photoreceptor degeneration

## Abstract

The asparaginase and isoaspartyl peptidase 1 (ASRGL1) is an L-asparaginase and beta-aspartyl peptidase enzyme that may be involved in the formation of L-aspartate, a neurotransmitter that can operate as an excitatory neurotransmitter in some brain regions. Although variants in *ASRGL*1 have been reported in retinitis pigmentosa (RP) patients, the *in vivo* functions and mechanisms of *ASRGL* in RP remains unknown due to the lack of suitable disease models. To explore the role of *ASRGL* in RP, we generated an *Asrgl1* knockout mouse model (*Asrgl1* KO) using the CRISPR/Cas9 technique. *Asrgl1* ablation in mice led to an attenuated electroretinogram (ERG) response around 8 months. The thickness of the outer nuclei layer (ONL) started to decrease around 9 months in *Asrgl1* KO mice and gradually intensified at 12 and 15 months. Immunostaining revealed thinner inner segment (IS) and thinner outer segment (OS) as well as the progressive degeneration of rod and cone cells in *Asrgl1* KO mice. One hundred forty-nine transcriptional differentially expressed genes (DEGs) were found by RNA-seq in *Asrgl1* KO retina. These DEGs were linked to a number of biological processes that were considerably enriched, including gastrointestinal disease and organismal injury and abnormalities. By analysis of canonical pathways, glucocorticoid receptor signaling was the most significant canonical pathway altered in *Asrgl1* KO retina. Several molecules, including NFE2L2, IL-4, Foxp3, and Fos, were in the central nodes of the interaction network in *Asrgl1* KO retina. In summary, our study provided a knockout mouse model for a better understanding of the molecular mechanism for *ASRGL1*-related RP.

## Introduction

Retinitis pigmentosa is one of the most common causes of visual impairment around the world, including symptoms such as night blindness and progressive loss of peripheral vision in the early stage, and no complete yet effective treatment exists up to date (Ferrari et al., 2011). The prevalence of this disorder is approximately 1:4,000 (Hartong et al., 2006). As an inherited retinal degeneration disease, it can be inherited in three ways: autosomal recessive (50%–60%), autosomal dominant (30%–40%), or X-linked (5%–15%). The phenotype of non-syndromic retinitis pigmentosa (RP) in RP patients might be limited to the eye or it can be part of a syndrome that includes extraocular illnesses such hearing loss, obesity, and neurologic diseases (Bhatti, 2006). Over 90 genes have been associated with non-syndromic RP according to the RetNet database (https://sph.uth.edu/retnet/; date last accessed Jul 30, 2021). However, only about 50%–60% of RP cases can be explained by these mutations, and the pathological and molecular mechanisms of some of the genes remain unknown (Daiger et al., 2013; Huang et al., 2015).

Asparaginase and isoaspartyl peptidase 1 (*ASRGL1*) has been reported as a RP disease gene in 2016 (Biswas et al., 2016). The p.G178R mutation, which causes photoreceptor degeneration and progressive vision loss, was discovered in a large five-generation pedigree with early-onset recessive retinal degeneration utilizing linkage analysis and homozygosity mapping combined with exome sequencing. *ASRGL1* encodes an enzyme L-asparaginase, has both L-asparaginase and beta-aspartyl peptidase activity, and may be involved in the production of L-aspartate (Cantor et al., 2009). However, the pathological and molecular mechanisms of *ASRGL1* in causing of RP remains unknown.

To investigate the function of *Asrgl1* in the mammalian retina and explore the molecular mechanism how *Asrgl1* affect photoreceptors or RPE cells, we developed a novel mouse model of RP with the *Asrgl1* gene knockout on a C57BL/6J genetic background by CRISPR/CAS9 technology. *Asrgl1* ablation in mice resulted in a typical retinal degeneration phenotype with decreased electroretinogram (ERG) response; thinner outer nuclei layer (ONL), inner segment (IS), and outer segment (OS); and decreased rod and cone proteins. A high-throughput transcriptional sequencing analysis found 149 transcriptional differentially expressed genes (DEGs) between *Asrgl1* knockout (KO) mouse retina and the normal controls. Glucocorticoid receptor signaling was the most changed pathway and might play key roles in the pathological process of *ASRGL1*-related RP. Our studies provide an *Asrgl1* KO mouse model for improving our understanding of RP disease mechanisms.

## Materials and Methods

### Generation of *Asrgl1* Knockout Mice

The Animal Care and Use Committee of the Sichuan Provincial People’s Hospital authorized all animal experiments, which followed the ARVO guidelines for the use of animals in ophthalmology and vision research.

**FIGURE 1:**
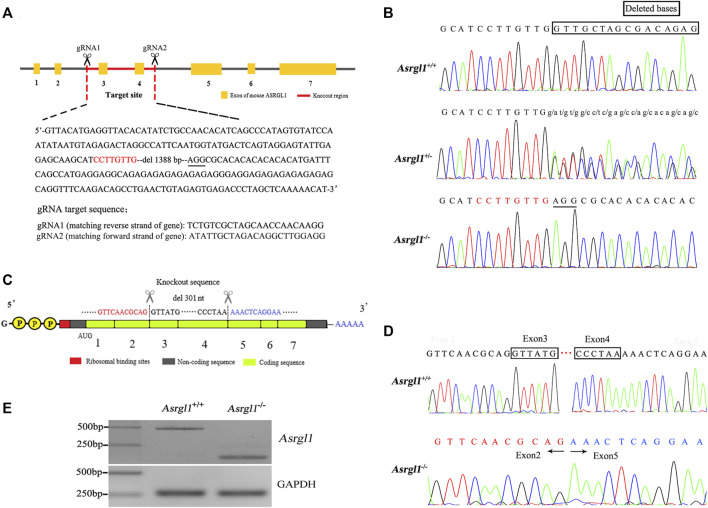
Generation of *Asrgl1* knockout (KO) mice. **(A)** The partial genomic sequence of *Asrgl1* exons was shown. The 1,388-bp genomic deletion was indicated in red. **(B)** Sanger sequencing of *Asrgl1* genome in the wild-type (WT), *Asrgl1* ^+/−^ and *Asrgl1* ^−/−^ mice. **(C)** Schematic illustration of cDNA deletion in *Asrgl1* KO mice. **(D)** Sanger sequencing of *Asrgl1* cDNA in WT and Asrgl1^−/−^ mice. A 301-bp fragment consisting of exon 3 and 4 is deleted in *Asrgl1* KO allele. **(E)** Reverse transcription PCR analysis of *Asrgl1* expression in WT and *Asrgl1* ^−/−^ mouse retinas at 3 months of age. Gapdh was used as a loading control.

*Asrgl1* KO mice were generated by using the CRISPR/Cas9 system on C57BL/6J genetic background. The Cas9 mRNA and two single guide RNAs targeting a region upstream of exon 3 and downstream of exon 4 of *Asrgl1* exon 3 were microinjected into mouse zygotes (Figure 1). gRNA1 sequence (matching reverse strand of gene) was as follows: TCT​GTC​GCT​AGC​AAC​CAA​CAA​GG. gRNA2 sequence (matching forward strand of gene) was as follows: ATA​TTG​CTA​GAC​AGG​CTT​GGA​GG. As confirmed by Sanger sequencing and PCR genotyping analysis, the conventional translation start site was successfully eliminated.

### Electroretinograms in Mice

Mouse ERG detection was performed on an Espion Visual Electrophysiology System (Diagnosys, Lowell, MA, United States). Briefly, female *Asrgl1* KO mice and female controls were dark-adapted overnight 1 day before detection. In the next morning, before ERG, animals were anesthetized and the eyes were dilated with a drop of tropicamide and phenylephrine, as well as tetracaine (0.5%). Throughout the experiment, a heating platform was used to keep the body temperature at 37°C. Gold wire loops were used to record dark-adapted ERGs in response to flashes with intensities ranging from 0.003 to 20 cds/m^2^. After 20 min of complete light adaptation, cone-mediated ERGs were recorded with white flashes.

### Retinal Hematoxylin and Eosin Staining

The eyes with the whole retina and optic nerve were chosen for imaging by hematoxylin and eosin (H&E) staining. Female wild-type (WT) and female KO mice’s eyes were enucleated, marked for orientation on the nasal side, fixed overnight in 1.22% glutaraldehyde and 0.8% paraformaldehyde in 0.08 M phosphate buffer, embedded in paraffin, and cut into 5-μm slices. H&E staining was used on sections taken from five places around the optic nerve. The rows of photoreceptors in the outer nuclear layer were counted using H&E-stained slices. Every 200 μm from the optic nerve, three measurements of the outer nuclear layer were recorded and averaged. The optic nerve was given the number 0 mm.

### Immunohistochemistry Analysis

Enucleated eyeballs were removed from female WT and female *Asrgl1* KO mice for immunohistochemistry, marked on the nasal side for orientation, fixed for 1 h in 4% paraformaldehyde in 100 mM phosphate buffer (pH 7.4), and cryoprotected in 30% sucrose. For sectioning, tissues were immersed in an optimal cutting temperature (OCT) solution and frozen on dry ice. For 30 min, sections were blocked and permeabilized in phosphate buffer with 10% normal goat serum and 0.2% Triton X-100, then labeled with different primary antibodies overnight at 4°C. Then, the slides were washed and incubated with secondary antibodies for 2 h. Finally, the slides were washed and covered with coverslips. The primary antibodies used in our study were as follows: rhodopsin (1D4, cat. #MA1-722, Thermo Fisher, Waltham, MA, United States), Na-K pump (cat. #MA3928, Thermo Fisher, Waltham, MA, United States), opsin (red/green, cat. #AB5405, Millipore, Burlington, MA, United States), and 594-conjugated peanut agglutinin (PNA) (cat. #RL1072, Vector laboratories, Burlingame, CA, United States). Secondary antibodies (Alexa Fluor 488 and Alexa Fluor 594) and DAPI (cat. #D8417, Sigma, St Louis, MO, United States, 1:2,000 dilution) were also used in the experiments. Confocal microscopy was used to image eye sections (LSM800, Carl Zeiss, Jena, Germany).

### RNA Isolation and RT-PCR

TRIzol (Invitrogen, Austin, TX, United States) was used to extract total RNA from the retinas of female *Asrgl1* KO mice and female normal controls, according to the manufacturer’s protocol. cDNA was made as previously described. An Eppendorf Mastercycler personal PCR equipment (Eppendorf, Germany) was used to amplify *Asrgl1* genotyping products, which were then analyzed on a 1.5% agarose gel electrophoresis. Using the SYBR PCR Master Mix kit (Applied Biosystems, Foster City, CA) and the 7500 Fast Real-Time PCR detection machine, equal amounts of cDNA from the retinas of *Asrgl1* KO mice and normal controls were submitted to PCR. Specific primers used in our study were listed in Supplementary Data S1.

### RNA-Seq and Differential Expression Analysis

RNA was isolated from the retinas of female *Asrgl1* KO mice and female normal controls, and the integrity of the RNA was determined using the Bioanalyzer 2100 system’s RNA Nano 6000 Assay Kit (Agilent Technologies, CA, United States). The library was then produced and tested for transcriptome sequencing using the Agilent Bioanalyzer 2100 instrument. The index-coded samples were clustered using the TruSeq PE Cluster Kit v3-cBot-HS (Illumina) on a cBot Cluster Generation System according to the manufacturer’s instructions. The library preparations were sequenced on an Illumina Novaseq device after cluster creation, yielding 150-bp paired-end reads. Raw data (raw readings) in fastq format were processed using in-house perl scripts, resulting in clean, high-quality data. The level of gene expression was measured after reads were mapped to the reference genome. All of the downstream analyses were based on clean, high-quality data. Prior to differential gene expression analysis, the read counts for each sequenced library were modified by one scaling normalization factor using the edgeR tool package. The edgeR R package was used to perform differential expression analysis of two situations. The *P* values were adjusted using the Benjamini and Hochberg method. The criterion for significant differential expression was set at 0.05 corrected *P*-value and log2 fold change. The raw data have been uploaded to the Genome Sequence Archive (https://ngdc.cncb.ac.cn/gsa/browse/CRA005193), and the assigned accession number was CRA005193.

### Functional Analysis, Canonical Pathway Analysis, and Generation of Networks

Top biological functions as well as canonical pathways associated with DEGs generated from the RNA-seq between the *Asrgl1* KO retina and normal controls were identified by Ingenuity pathway analysis (IPA) (Qiagen). Fisher’s exact test was used to determine the likelihood of any biological function or canonical pathway being explained only by chance. Algorithmically, molecular interaction networks are created based on the connectivity of the molecules. Fisher’s exact test was used to determine network scores, which corresponded to −log 10 (*p* value).

### Statistical Analysis

**FIGURE 2:**
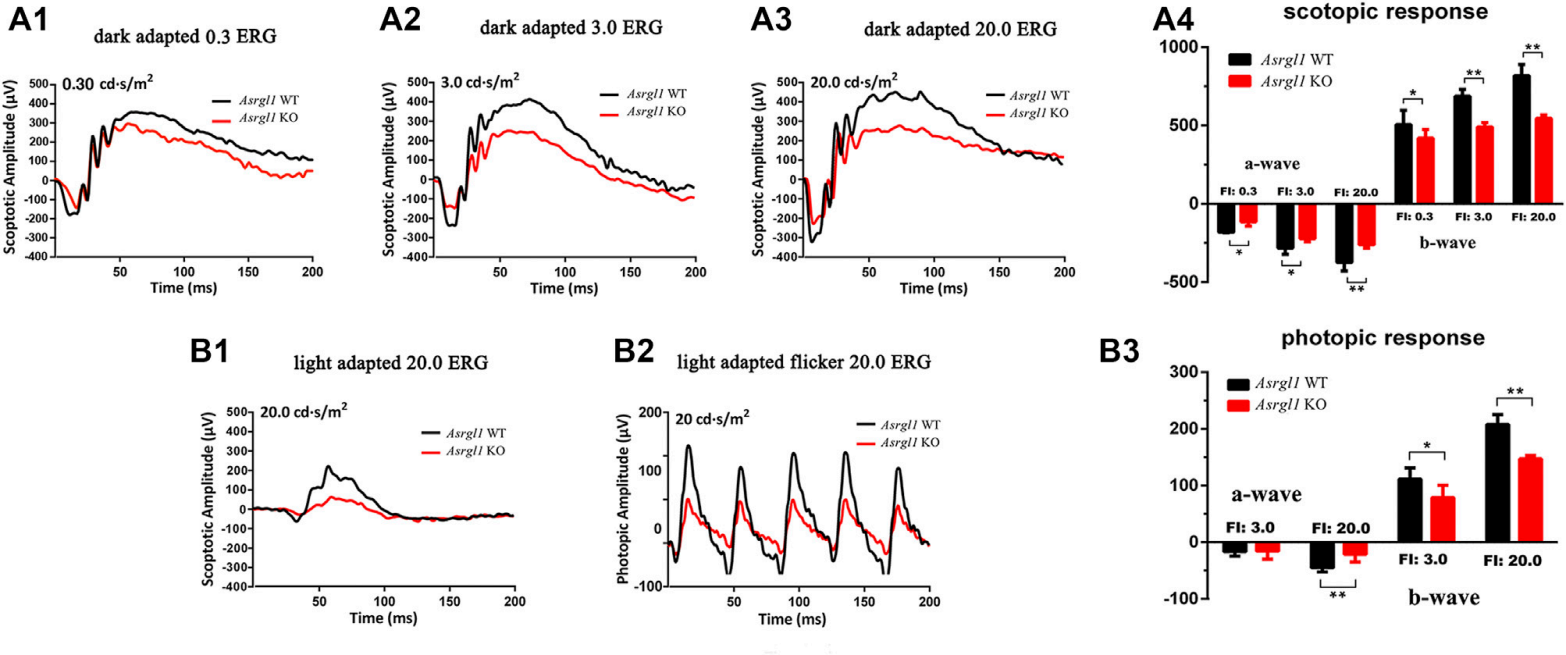
ElectroretinogramERG) examinations of *Asrgl1* KO mice. **(A1–A4)** Asrgl1 KO mice at 8 months of age exhibited a reduced ERG scotopic response. Reduced amplitude peaks were observed for both the *a*-wave and the *b*-wave at light intensities of 0.3, 3.0, and 20.0 cds/m^2^ in *Asrgl1* KO mice; two-tailed Student’s *t*-test was used. ∗∗*p* < 0.01; ∗∗∗*p* < 0.001. **(B1–B3)** Compared with WT mice, 8-month-old KO mice exhibited a reduced photopic response at 20.0 cds/m^2^; sample size *n* = 4. A two-tailed Student’s *t*-test was used. ∗∗*p* < 0.01; ∗∗∗*p* < 0.001.

**FIGURE 3:**
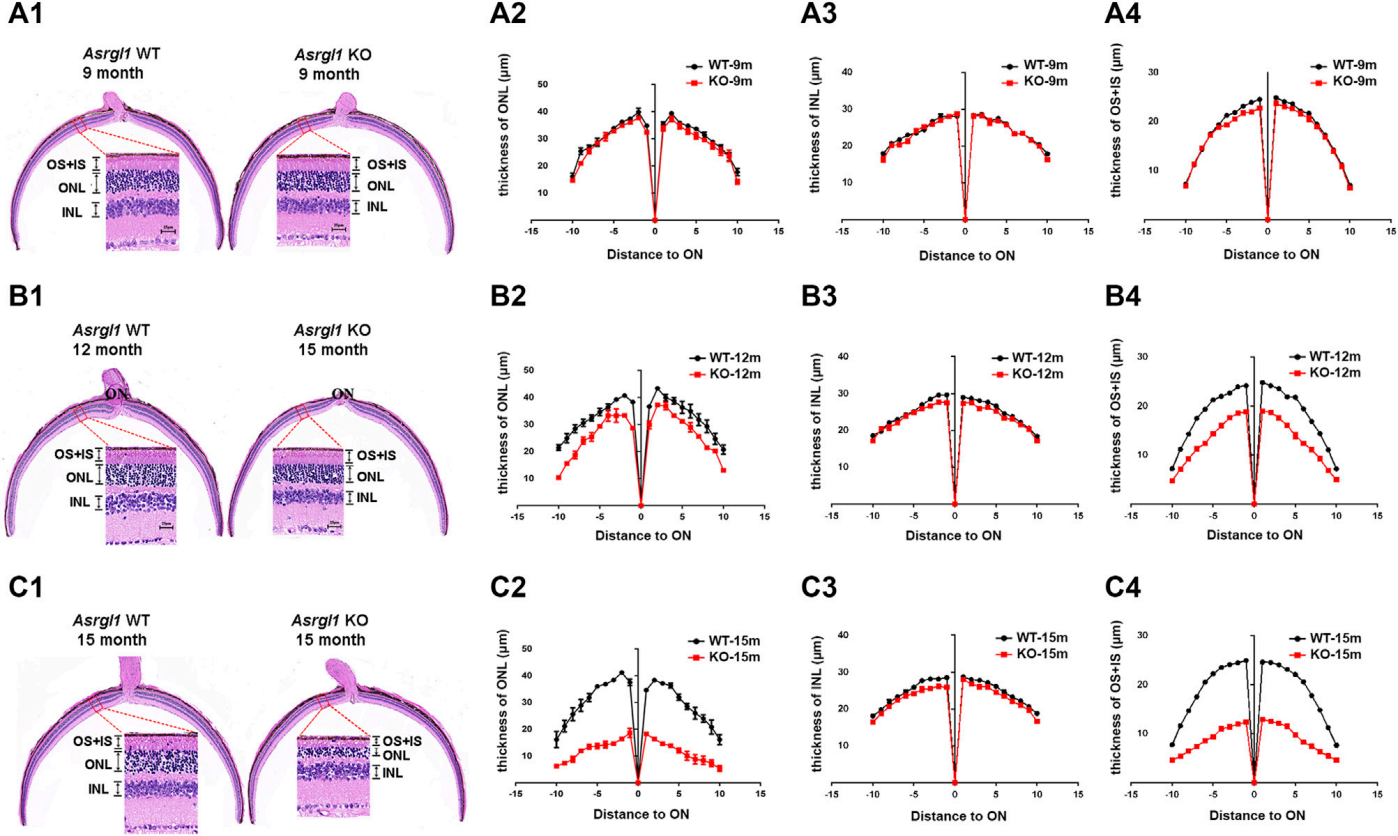
Degeneration of the outer retina in *Asrgl1* KO mice. **(A1,B1,C1)** Hematoxylin and eosin (H&E) staining of paraffin sections of the *Asrgl1* KO and corresponding control retinas at the ages of 9, 12, and 15 months. Scale bar: 25 μm. **(A2,B2,C2)** Quantification analysis of the outer nuclei layer (ONL) thickness of the KO (*n* = 6) and WT (*n* = 6) retinas from mice at 9, 12, and 15 months old. **(A3,B3,C3)** Quantification analysis of the inner nuclei layer (INL) thickness of the KO (*n* = 6) and WT (*n* = 6) retinas from mice at 9, 12, and 15 months old. **(A4,B4,C4)** Quantification analysis of the outer segment (OS) plus inner segment (IS) thickness of the KO (*n* = 6) and WT (*n* = 6) retinas from mice at 9, 12, and 15 months old. The *Asrgl1* KO mouse retinas showed a drastically gradual degeneration of the photoreceptor cells at 15 months old.

Statistical significance was established using Prism 7.0 software and the Student’s *t*-test or one-way ANOVA (GraphPad Software, La Jolla, CA, United States). The data are presented as the mean SEM unless otherwise noted in the text and figure legends. The ERG datasets in Figure 2 and the ONL thickness measurement dataset in Figure 3 were subjected to ANOVA tests. The number of animals used in each experiment is indicated by *n*. In the figure legends, the total number of animals used in each experiment is also listed. The following *p* values are indicated by asterisks in the figures: *≤0.05, **≤0.01, and ***≤0.001.

## Results

### Generation of *Asrgl1* Knockout Mice

ASRGL1 is widely expressed in all human tissues studied, with the highest levels of expression in the brain, female tissues including the uterine cervix and fallopian tube, and male tissues like the testis (Edqvist et al., 2015). To investigate the functions of *AsrgL1 in vivo*, *Asrgl1* KO were generated using CRISPR/Cas9 technology. The target sites were selected as exon 3 and exon 4 (Figure 1A). Genotyping analysis using PCR primers flanking the deletion site revealed the successful generation of *Asrgl1* knockout mice (Supplementary Figure S1). To validate the genomic *Asrgl1* ablation, mouse DNA samples were subjected to Sanger sequencing analysis, and results showed genomic deletion of 1,388 bp in *Asrgl1* KO mice (Figure 1B). Both Sanger sequencing and electrophoresis analysis of cDNA product from *Asrgl1* KO retinas revealed a 301-bp deletion of exon 3 and exon 4 in *Asrgl1* KO (Figure 1C–E). All the above data showed the successful generation of *Asrgl1* KO by CRISPR/Cas9 technology.

### *Asrgl1* Knockout Mice Exhibited Impaired Visual Function

ERG examination was first performed to test the *in vivo* function of *Asrgl1* in the retina. In three 6-month-old *Asrgl1* KO mice and littermate controls, no abnormal ERG response was observed (data not shown). However, all *Asrgl1* KO mice exhibited reduced scotopic and photopic responses at 8 months of age. The mean *a*- and *b*-wave amplitudes of *Asrgl1* KO were reduced by approximately 15% and 30%, respectively, in scotopic response tests (Figure 2A1–A4), which indicated altered rod cell function. When compared to WT mice, *Asrgl1* KO had a somewhat flat wave under light-adapted conditions, showing that cone cell function was also impaired in *Asrgl1* KO (Figure 2B1–B3). Although, by H&E staining, the retinas from *Asrgl1* KO mice revealed no difference in the retinal ONL thickness at 6 months of age (Supplementary Figure S2), the thickness of the ONL started to decrease around 9 months of age (Figure 3A1, A2) and gradually intensified at 12 months (Figure 3B1, B2) and 15 months of age (Figure 3C1, C2). The photoreceptor layer including the inner segment and outer segment decreased around 9 months (Figure 3A1, A4) and intensified at 12 months (Figure 3B1, B4) and 15 months of age (Figure 3C1, C4). There was no obvious change in the inner nuclei layer (INL) of *Asrgl1* KO at 9 months of age (Figure 3A1, A3), and the thickness of the INL in *Asrgl1* KO was slightly decreased at 15 months of age (Figure 3C1, C3).

### Loss of *Asrgl1* Leads to Degeneration and Apoptosis of Photoreceptor Cells

**FIGURE 4:**
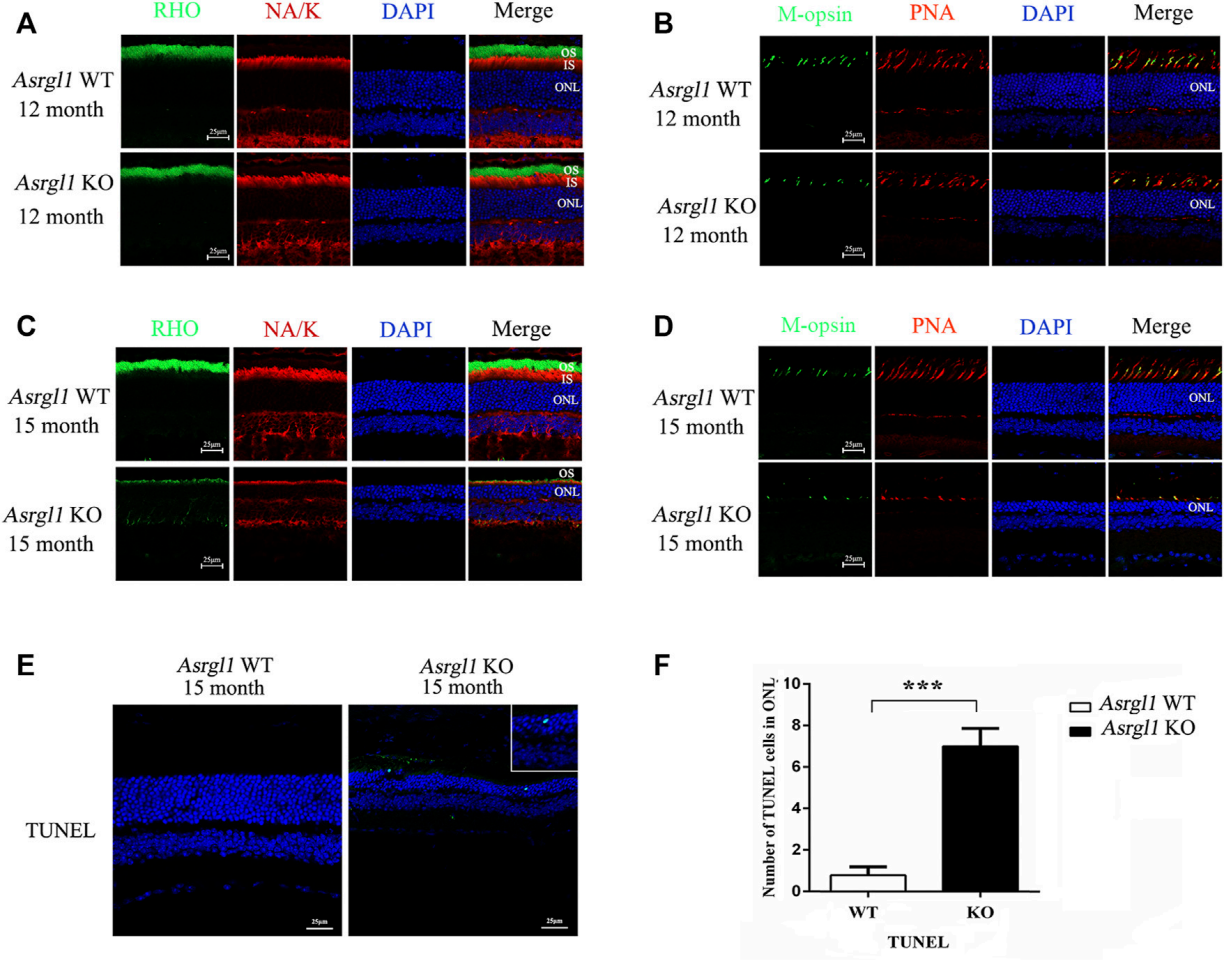
Immunofluorescence staining analysis of the *Asrgl1* KO retinas. **(A,C)** Retinal cryosections from 12- and 15-month-old mice (*n* = 6) were labelled with rod-specific markers rhodopsin (outer segment) and Na-K ATPase (inner segment). **(B,D)** M-opsin and peanut agglutinin (PNA) was used as the cone markers in 12- and 15-month-old mice (*n* = 6). DAPI was used to counterstain the nuclei; scale bars: 25 μm. The data shown are representative of three independent tests. At least three slides of each retina were stained. **(E)** TUNEL assays in 15-month-old WT and KO mice revealed increased apoptosis. The magnified square indicated TUNEL-positive cells in the retinas. This experiment was performed three times. Sample size *n* = 6. **(F)** Quantification of TUNEL-positive cells in WT and KO retinas; sample size *n* = 6; ∗∗∗*p* < 0.001. Statistical significance was determined by a two-tailed unpaired *t*-test.

To investigate the pathological changes underlying the *Asrgl1* knockout mice’s retinal degeneration phenotype, immunostaining was performed with retinal cryosections in *Asrgl1* KO and WT mice. There were no changes in rhodopsin and M-opsin expression in *Asrgl1* KO at 8 months of age (Supplementary Figure S3A, B). However, decreased rhodopsin protein was observed in the photoreceptor of 12- and 15-month-old *Asrgl1* KO mice, respectively (Figure 4A, C), compared to WT mice, which indicated a shortened outer segment of 12- and 15-month-old *Asrgl1* KO mice. Rhodopsin is a key outer segment protein that is required for adequate vision, and its manufacture and transit are tightly regulated (Palczewski, 2006). Decreased rhodopsin is cytotoxic and leads to rod death. Decreased Na-K ATP staining in 15-month-old *Asrgl1* KO mice indicated shortened inner segment. M-opsin, a cone cell marker, and PNA (which binds to the sheaths of the cone matrix in all three types of cones) were also used to stain retinal sections. The number of cone cells was reduced in 12-month-old *Asrgl1* KO mice and drastically decreased in 15-month-old *Asrgl1* KO mice (Figure 4B,D). A TUNEL assay was utilized to determine whether the retinal degeneration was caused by apoptosis. There were no changes in TUNEL-positive cells in 8-month-old *Asrgl1* KO mice (Supplementary Figure S3C). TUNEL-positive cells were substantially more abundant at 15 months of age in *Asrgl1* KO retinas than in WT retinas (Figure 4E,F), and TUNEL-labeled nuclei were mostly found in the ONL, indicating continuous photoreceptor cell death in Asrgl1 KO retinas.

### Differentially Expressed Genes in the Retinas Between *Asrgl1* KO and Normal Controls

**FIGURE 5:**
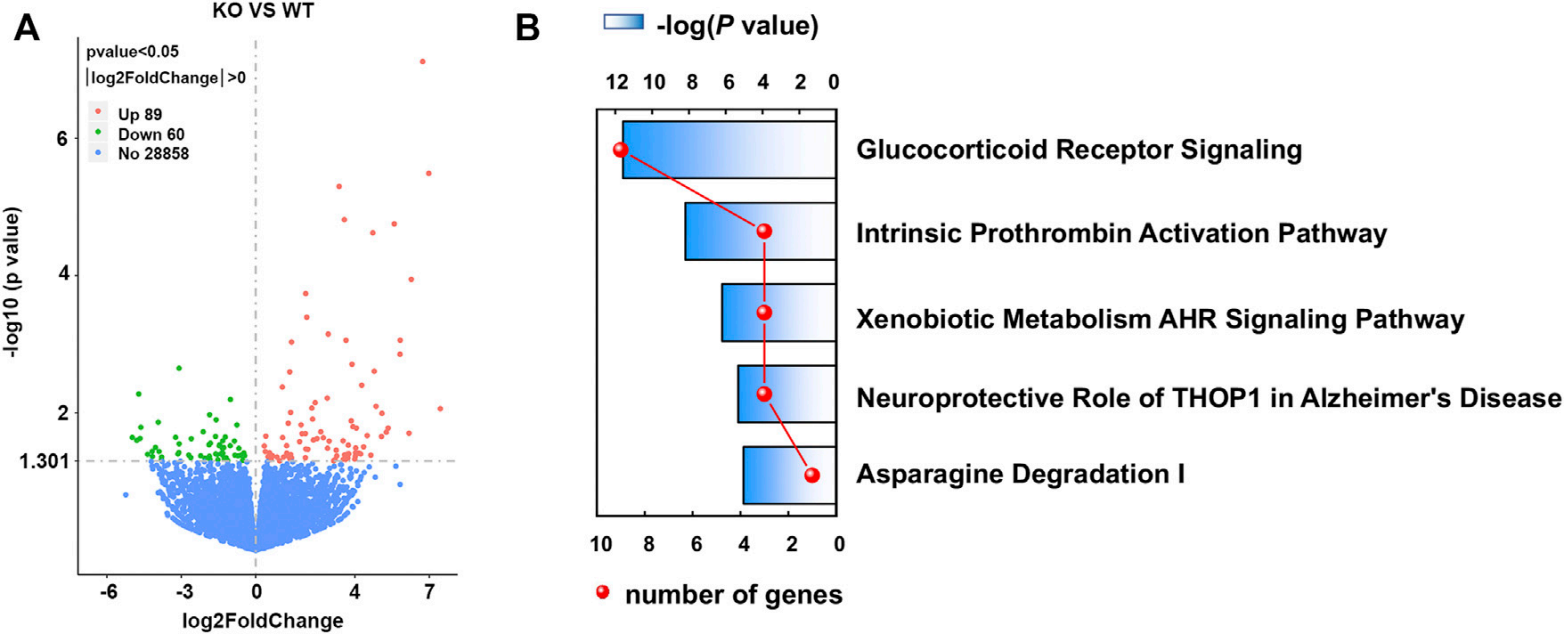
Volcano plots of differentially expressed genes (DEGs) and top canonical pathways involved in *Asrgl1* KO mice. **(A)** Volcano plots of DEG profile. The vertical lines correspond to 2.0 FC up and down, and the horizontal line represents −log 10 (*P*-value). **(B)** Ingenuity pathway was used to analyze the total significantly differentially expressed genes and identify the canonical pathways involved in *Asrgl1* KO mice. The top five significant pathways identified were displayed (blue bar). The red curve shows the total number of genes in each of these pathways.

To investigate the transcriptional effect of *Asrgl1* ablation, mouse retinal tissues from 8-month-old *Asrgl1* KO (*n* = 3) and WT mice (*n* = 4) were subjected to RNA-seq analysis. There were 149 DEGs identified in *Asrgl1* mice (Supplementary Table S2), among which 89 were upregulated (*p* ≤ 0.01 and log2 ratio ≥1) and 60 (*p* ≤ 0.01 and log2 ratio ≤−1) were downregulated. Volcano plots of differentially expressed genes are shown in Figure 5A. IPA was used to identify numerous highly enriched biological processes related with DEGs in order to investigate the biological functions enriched in DEGs (Table 1). Gastrointestinal disease (*p* value: 4.79E−02 to 2.61E−05, 13 genes assigned), organismal injury and abnormalities (*p* value: 4.94E−02 to 2.61E−05, 31 genes assigned), and cancer (*p* value: 4.79E−02 to 3.23E−04, 18 genes assigned) were discovered as the top three highly enriched terms in the perspective of diseases and disorders. Cellular development (*p* value: 4.16E−02 to 3.14E−05, seven genes assigned), cell morphology (*p* value: 4.79E−02 to 1.07E−03, six genes assigned), and cell death and survival (*p* value: 4.94E−02 to 1.22E−03, seven genes assigned) were found as the top three highly enriched terms in the perspective of molecular and cellular functions. Digestive system development and function (*p* value: 3.38E−02 to 2.61E−05, seven genes assigned), organ morphology (*p* value: 4.48E−02 to 2.61E−05, 13 genes assigned), and hematological system development and function (*p* value: 4.60E−02 to 3.14E−05, seven genes assigned) were shown as the top three enriched terms in the perspective of physiological system development and functions.

**TABLE 1:** Functional analysis for the DEGs in the retina between *Asrgl1* knockout mice and normal controls.

Top diseases and bio functions	*p*-value range	# of molecules
Diseases and disorders		
Gastrointestinal disease	4.79E−02–2.61E−05	13
Organismal injury and abnormalities	4.94E−02–2.61E−05	31
Cancer	4.79E−02–3.23E−04	18
Dermatological diseases and conditions	4.48E−02–1.07E−03	8
Reproductive system disease	3.85E−02–1.32E−03	8
Molecular and cellular functions		
Cellular development	4.16E−02–3.14E−05	7
Cell morphology	4.79E−02–1.07E−03	6
Cell death and survival	4.94E−02–1.22E−03	7
Cellular growth and proliferation	4.16E−02–1.22E−03	5
Amino acid metabolism	19.76E−03–3.26E−03	1
Physiological system development and function		
Digestive system development and function	3.38E−02–2.61E−05	7
Organ morphology	4.48E−02–2.61E−05	13
Hematological system development and function	4.60E−02–3.14E−05	7
Lymphoid tissue structure and development	1.94E−02–3.14E−05	2
Hair and skin development and function	4.48E−02–1.07E−03	3

a Range of p-values indicate higher-level functions that contained multiple lower-level functions.

### Glucocorticoid Receptor Signaling Pathway Is Involved in *Asrgl1* Knockout Mice

Canonical pathway analysis could inform the key metabolism and signaling pathways in which the DEGs may be involved. The main metabolism and signaling pathways in which DEGs may be engaged could be informed *via* canonical pathway analysis. In this study, canonical molecular pathways enriched by DEGs were explored by bioinformation analysis of IPA software. Figure 5B shows the top five canonical pathways in the retina of *Asrgl1* KO mice that are significantly associated with DEGs. The glucocorticoid receptor signaling (*p* value = 9.36E−06), intrinsic prothrombin activation pathway (*p* value = 2.78E−04), and xenobiotic metabolism AHR signaling pathway (*p* value = 2.04E−03) were the top three major pathways altered in retinal tissues in *Asrgl1* KO mouse. The molecules that were changed in the glucocorticoid receptor signaling were IL1β, KRT5, KRT7, KRT13, KRT14, KRT15, KRT19, KRT80, and KRT6B. Our results firstly suggested an association of *Asrgl1* with GR signaling in the pathological process of retinitis pigmentosa.

### Influenced Interaction Network by Ablation of *Asrgl1* in Mouse Retina

**FIGURE 6:**
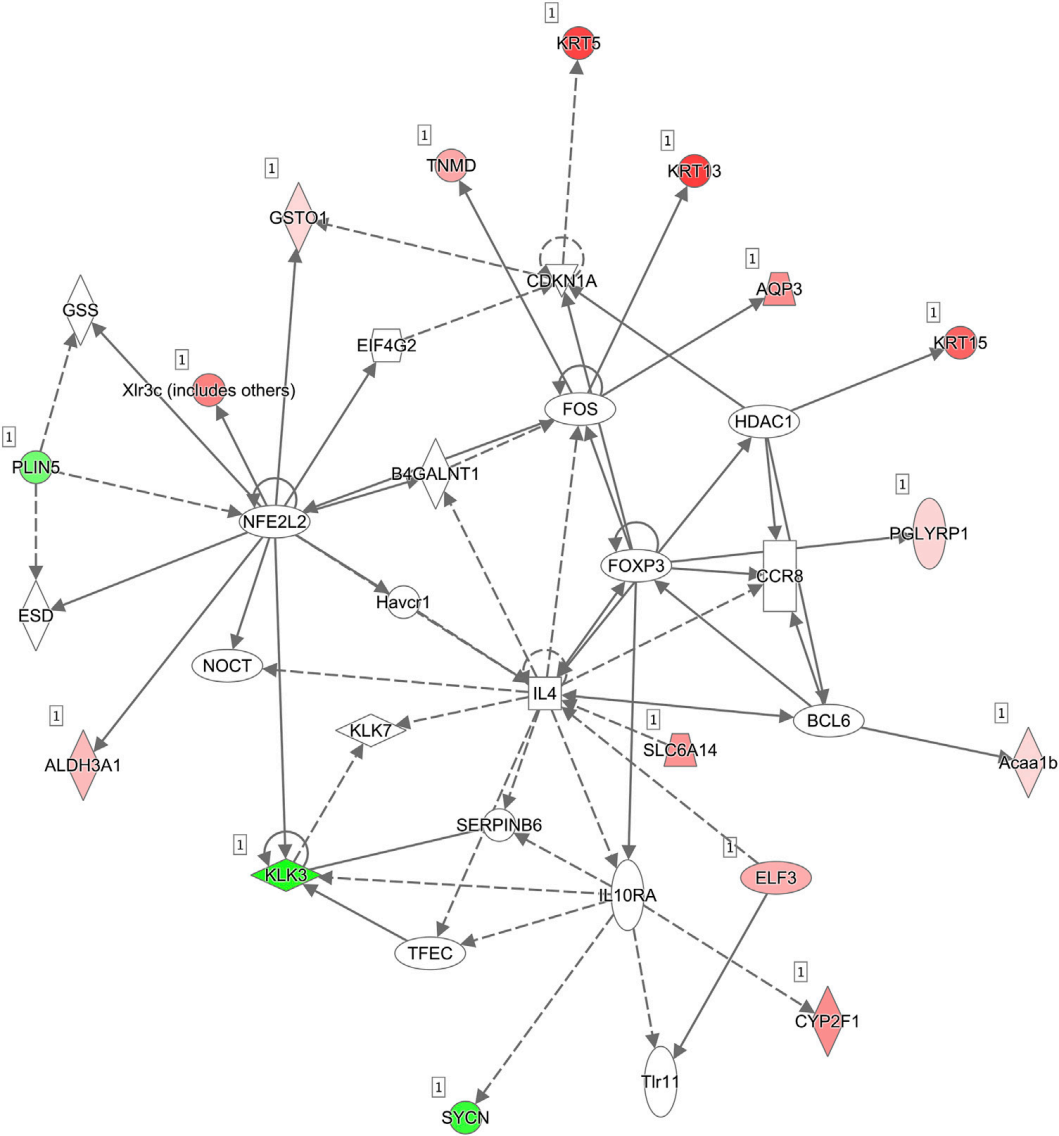
Molecular interaction networks. The most significant biological network of “Cell Morphology, Embryonic Development” was generated. Upregulated mRNAs are indicated in red, while downregulated mRNAs are in green. Solid lines represent the direct function, while the dotted lines represent the indirect function.

**FIGURE 7:**
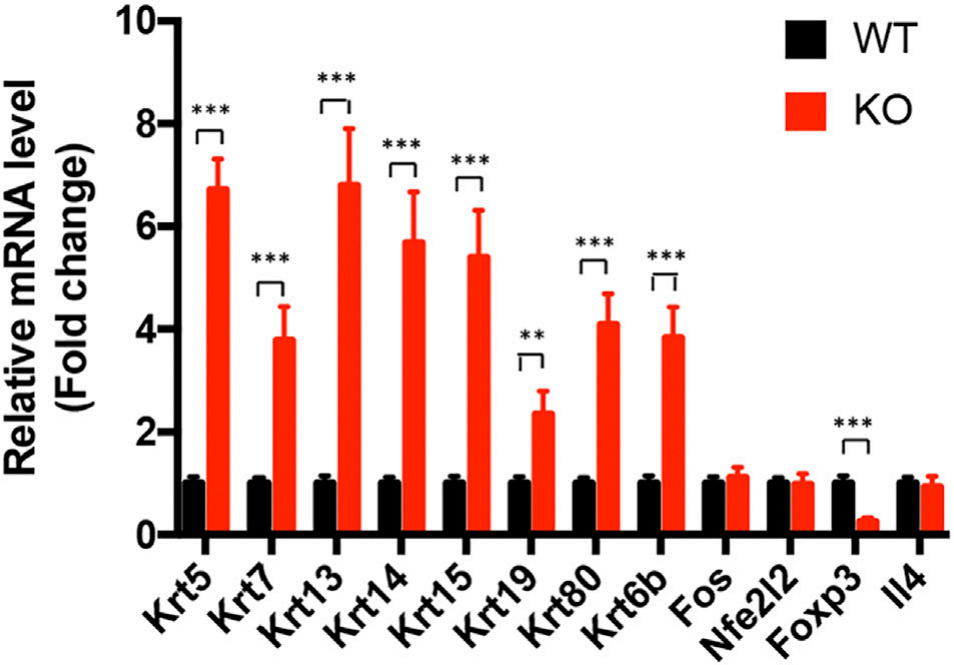
Transcriptional validation of the differentially expressed genes in *Asrgl1* KO mice. The expression of Krt5, Krt7, Krt13, Krt14, Krt15, Krt19, and Krt80 in glucocorticoid receptor signaling pathway and the “central node” genes, NFE2L2, IL-4, Foxp3, and Fos, in the interaction network between WT and *Asrgl1* KO mice (*n* = 6) was validated by real-time PCR. A two-tailed Student’s *t*-test was used; ∗∗*p* < 0.01; ∗∗∗*p* < 0.001. Data were from an average of four samples per group. Each test was repeated at least three times.

The molecular interaction networks were then created and ranked based on the connectivity of the detected DEGs. The network of “Cell Morphology, Embryonic Development” (score = 49) (Figure 6) was the most enriched network alerted in retinal tissues of *Asrgl1* KO mice. Notably, several genes including NFE2L2, IL-4, Foxp3, and Fos were the core “nodes” with the most connections in the network. To validate the expression levels of the main altered genes in glucocorticoid receptor signaling pathway and network of “Cell Morphology, Embryonic Development,” real-time PCR was performed. As Figure 7 shows, the expression of KRT5, KRT7, KRT13, KRT14, KRT15, KRT19, and KRT80 in glucocorticoid receptor signaling pathway all increased in *Asrgl1* knockout mice compared to the normal controls. Interestingly, only the expression of Foxp3 was decreased among the selected “central node” genes, namely, NFE2L2, IL-4, Foxp3, and Fos. As Foxp3 is a transcriptional regulator that is required for regulatory T-cell development and inhibitory activity (Treg) (Li et al., 2015), its function in *Arsgl1*-related RP needs to be explored further in follow-up studies.

## Discussion

RP is the leading cause of inherited blindness in the developed world. Although more than 90 genes have been found associated to RP, some of the gene functions remain unknown (Dias et al., 2018). There are numerous RP animal models available, which have contributed to a better understanding of the disease’s pathogenesis and the development of therapeutic strategies targeted at curing or delaying the hereditary illness (Petersen-Jones, 1998). In this study, we generated a novel *Asrgl1* knockout mouse model to investigate the *in vivo* roles of *ASRGL1*, a new RP gene reported in 2016.

We found *Asrgl1* ablation led to an attenuated ERG response around 8 months. The thickness of the ONL started to decrease at around 9 months in *Asrgl1* KO mice and gradually intensified at 12 and 15 months. Thinner IS and OS as well as the diminished expression of functional markers in the rod and cone were revealed by immunostaining in *Asrgl1* KO mice. Although the patients exhibited an early-onset retinal dystrophy, late-onset retinal dystrophy was observed in *Asrgl1* KO mice. It is not uncommon for the phenotypes of people with *Asrgl1* mutations and those of knockout mice to differ. One example is the difference between patients with CRB1 mutations and retinal degeneration-8 (rd8) mice phenotypes. CRB1 mutations led to retinitis pigmentosa 12, LCA8, or childhood- and juvenile-onset cone–rod dystrophy (den Hollander et al., 2004; Bujakowska et al., 2012). The majority of the patients exhibited severe vision loss. However, Mehalow et al. (2003) described a new mouse model, rd8, with a single-base loss in the Crb1 gene in mice. rd8 mutation resulted in a frameshift and premature stop codon. The truncated protein lost the transmembrane and cytoplasmic domains of CRB1. However, rd8 homozygous mutant mice only exhibited mild retinal degeneration. Photoreceptor degeneration was observed only within spotted regions of the retina. Furthermore, the severity of retinal pathology is influenced by genetic backgrounds (Aleman et al., 2011; Luhmann et al., 2015). Thus, our *Asrgl1* knockout mouse model showed a late-onset photoreceptor degeneration phenotype, which is valuable to investigate the pathogenesis in *ASRGL1*-related RP and further assess the *in vivo* function of *Asrgl1* in the retina.

The 308-amino-acid ASRGL1 protein is activated by autocleavage at amino acid 168, forming an alpha- and beta-chain that can dimerize into a heterodimer (Cantor et al., 2009; Li et al., 2012). In some brain regions, the ASRGL1 enzyme is involved in the synthesis of L-aspartate, which can operate as an excitatory neurotransmitter. In endometrioid endometrial carcinoma, loss of *ASRGL1* expression was an independent biomarker for disease-specific survival, and combined *ASRGL1* and p53 could be used as an independent predictor of survival (Edqvist et al., 2015; Huvila et al., 2018). *Asrgl1* was reported to be trafficked by neural stem/progenitor cell (NSC)-derived extracellular vesicles (EVs) and thus enables NSC EVs as independent metabolic units with asparaginase activity (Iraci et al., 2017). Biswas et al. (2016) showed that the p.G178R mutation in *ASRGL1* impaired the autocatalytic processing of ASRGL1 and resulted in the function loss of *ASRGL1*, which caused early-onset recessive retinal degeneration in a five-generation pedigree. Although the authors observed retinal abnormalities and loss of cone photoreceptors in a zebrafish model, the *in vivo* function of *ASRGL1* in mammalian animals remains unknown. In our study, we firstly observed the photoreceptor degeneration in *Asrgl1* knockout mouse models as well as the transcriptional molecular changes in the *Asrgl1* knockout mouse retina.

Deletion of genomic fragments in *Asrgl1* knockout mice was performed by CRISPR/Cas9 technology. Several available antibodies against ASRGL1 were subjected to immunoblotting and immunostaining analysis. Unfortunately, none of them worked well. Since *Asrgl1* is consist of seven exons and encodes a 326-amino-acid protein in *Mus musculus*, the deletion of a 301-bp fragment spanning exon 3 and exon 4 in *Asrgl1* knockout mice most likely led to generation of shortened proteins lacking majority of the downstream domains after exon 2 and resulted in impaired formation of heterodimer comprising an alpha- and beta-chain by autocleavage.

To further reveal the molecular mechanism of *ASRGL1* in mouse retinas, we explored differentially expressed genes, pathways, and interaction network in *Asrgl1* knockout mouse retinas by transcriptional analysis. Our results suggested an important role of glucocorticoid receptor signaling in the physiology of *Asrgl1*-related retinal degeneration. Although glucocorticoid receptor (GR) has been found in various cell types of the eye, including the retina, little is known yet about the mechanism of glucocorticoid signaling in distinct layers of the eye (Kadmiel and Cidlowski, 2013; Gallina et al., 2014; Sulaiman et al., 2018). As GR signaling is commonly associated with anti-inflammatory responses, GR agonists are widely used to treat inflammatory diseases of the eye. Further investigation is warranted to explore how *ASRG1L* influence the glucocorticoid receptor signaling pathway and its role in the pathological process of RP. Interestingly, glucocorticoids have been reported to suppress the expression of a subset of the keratin family genes K5-K14, K6-K16, and K17 (Radoja et al., 2000). However, in the present study, several genes in the keratin gene family, such as Krt5, Krt7, Krt13, Krt14, Krt15, Krt19, and Krt80, were found to have increased transcriptional expression in the *Asrgl1* knockout mice. The keratin gene family consists the highest number of members in humans with 54 distinct functional genes (Moll et al., 2008). The functions of several keratins in the eye have been reported. Krt8/keratin 8 was shown to protect against degeneration of retinal pigment epithelium under oxidative stress (Baek et al., 2017), and KRT3 and KRT12 gene mutations associated with Meesmann corneal dystrophy (Chen et al., 2015). However, the function of keratin family genes in the retinas still needs to be further investigated. Several molecular interaction networks were generated using the identified DEGs. In the most enriched network, “Cell Morphology, Embryonic Development,” NFE2L2, IL-4, Foxp3, and Fos were the network’s central “nodes” with the greatest number of connections, which indicated the important roles of these molecules in the *Asrgl1* knockout retina. NFE2L2 (NRF2) is a transcription factor involved in the oxidative stress response, which has been linked to several ocular diseases (Ildefonso et al., 2016; Naguib et al., 2021). An anti-inflammatory cytokine, IL-4, has been reported to inhibit the proliferation of retinal cells (Silva et al., 2008). Foxp3 is a transcriptional regulator that is required for regulatory T-cell development and inhibitory activity (Treg). Foxp3+ Tregs can be recruited to the retina to repair abnormal angiogenesis (Deliyanti et al., 2017). C-fos has been linked to cell death and regeneration in retinal ganglion cells (Oshitari et al., 2002). Through c-Fos, inflammatory signals from photoreceptors influenced pathological retinal angiogenesis (Sun et al., 2017). Except the most enriched network “Cell Morphology, Embryonic Development,” several other interaction networks with lower scores were identified (not shown). Although, we revealed the transcriptional regulation of glucocorticoid receptor signaling pathway and several molecules in *Asrgl1* KO mice, the intrinsic mechanism will be further explored in our future work.

In conclusion, our study firstly explored the function of *Asrgl1* using a novel knockout mouse model and confirm its role in the pathological process of RP. Studies utilizing mouse models have been proven important not only in recapitulation of the disease phenotype in humans but also in improving our understanding of disease mechanisms, and this knockout model is valuable for further development of translational therapeutic approaches.

## Data Availability

The data presented in the study are deposited in the Genome Sequence Archive (https://ngdc.cncb.ac.cn/gsa/browse/CRA005193), accession number was CRA005193.
