# Supplementary figures and images for: Deletion of Asrgl1 Leads to Photoreceptor Degeneration in Mice
Source: Front Cell Dev Biol. 2022 Jan 18;9:783547. doi: 10.3389/fcell.2021.783547 (PMC8805730; doi:10.3389/fcell.2021.783547)

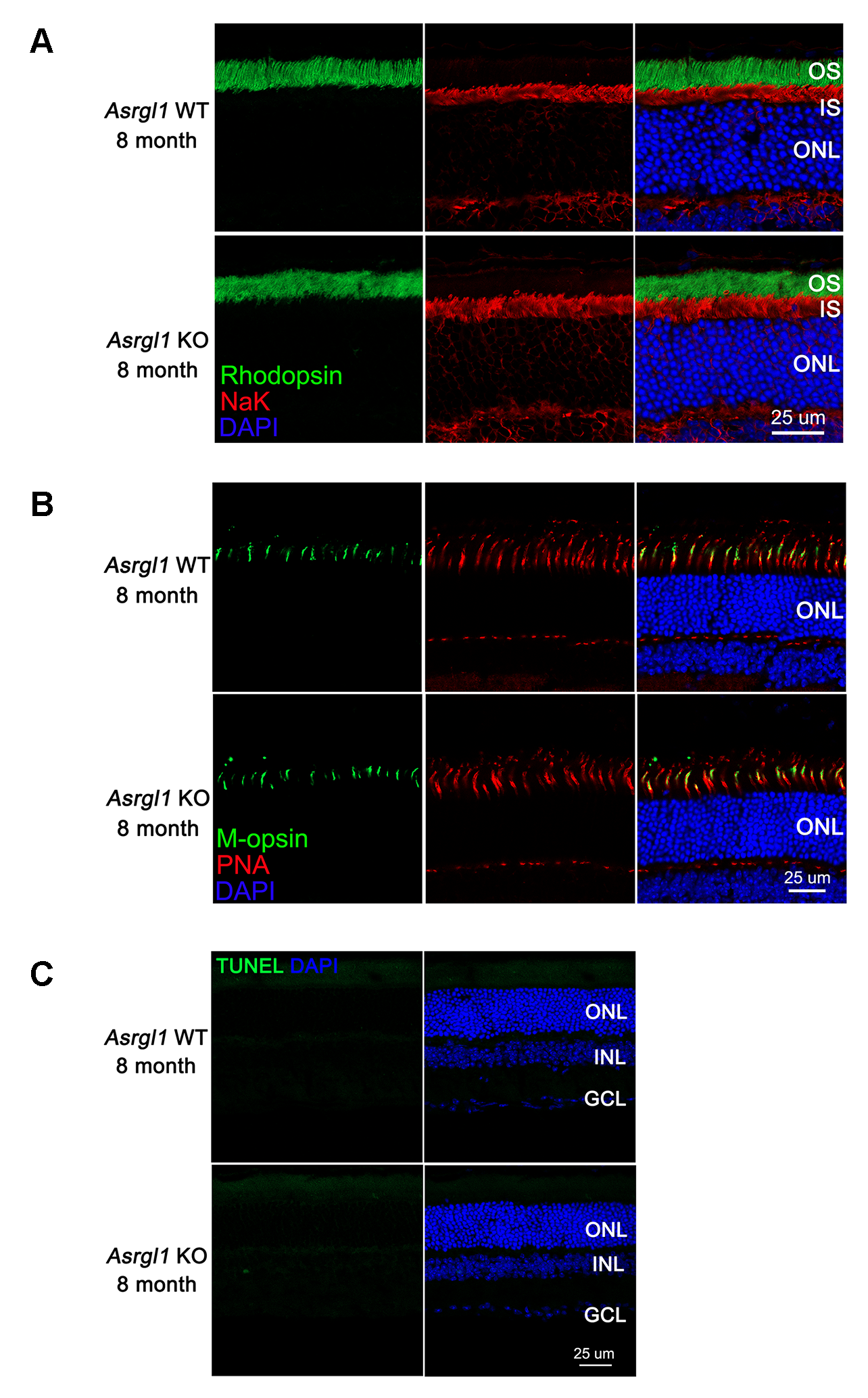

Supplement: Supplementary file 2 [file Image3.tif]

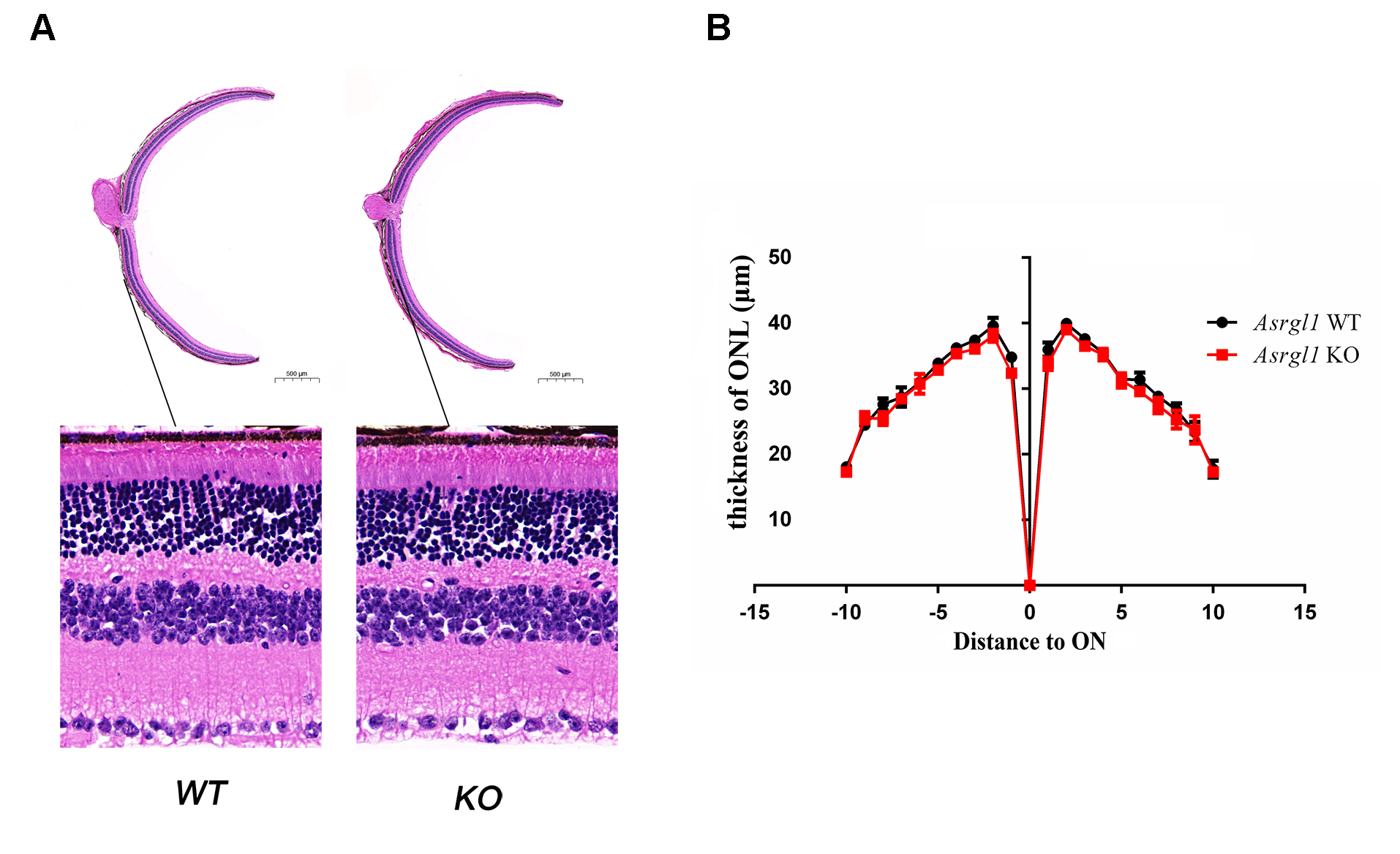

Supplement: Supplementary file 3 [file Image2.tif]

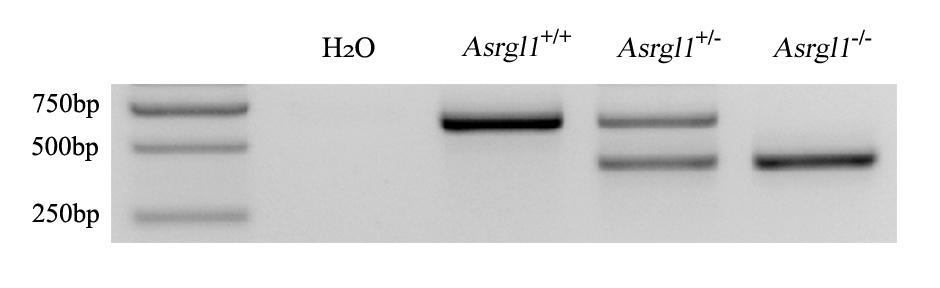

Supplement: Supplementary file 4 [file Image1.tif]
